# Contrasting Patterns of Plants, Bees, Hoverflies and Spiders in Different Habitats in a Central European Agricultural Landscape

**DOI:** 10.1002/ece3.70711

**Published:** 2024-12-16

**Authors:** Flóra Vajna, Raoul Pellaton, Csaba Molnár, Zoltán Soltész, Nikolett Gallé‐Szpisjak, Áron Domonkos Bihaly, András Báldi

**Affiliations:** ^1^ Lendület Ecosystem Services Research Group Institute of Ecology and Botany Vácrátót Hungary; ^2^ Freelance Researcher Basel Switzerland; ^3^ Freelance Researcher Gömörszőlős Hungary; ^4^ Lendület Landscape and Conservation Ecology Research Group Institute of Ecology and Botany, HUN‐REN Centre for Ecological Research Vácrátót Hungary; ^5^ Department of Zoology and Ecology, Institute for Wildlife Management and Nature Conservation Hungarian University of Agriculture and Life Sciences Gödöllő Hungary

**Keywords:** biocontrol, multifunctional landscape, nature restoration, pollination

## Abstract

Semi‐natural grasslands and their biodiversity decline rapidly, although they are key elements of agricultural landscapes. Therefore, there is a need for the re‐establishment of semi‐natural grasslands in intensively managed farmlands (e.g., via sowing wildflower seeds). Our knowledge, however, is limited on how different arthropod groups may respond to such newly established wildflower fields. This knowledge gap is especially relevant for the Pannonian biogeographical region, and more generally for Central Europe, where there is little to no evidence so far. We aimed to compare three different habitats (i.e., sown wildflower fields (WFF), semi‐natural road verges and adjacent crop fields) in terms of their species and individual numbers and assemblage compositions to reveal differences between primary producers (plants), pollinators (bees and hoverflies) and predators (spiders). We selected eight landscapes in Central Hungary within conventionally managed crop areas. We analysed species and individual numbers by generalised linear mixed models (GLMM) and the assemblage composition with non‐metric multidimensional scaling for each taxon in the three habitats. Crop, road verge and WFF habitats had distinct assemblages for each studied group, indicating clear separation among habitats. There are, however, contrasting patterns in the diversity measures of the studied groups. Crop fields are the poorest in both species and individual numbers, road verges harboured the highest abundance of spiders, while WFF had the most bees and plants. No clear pattern for hoverflies emerged. Our results suggest that the studied habitats do not harbour all groups in equal share. We propose that the design of future restorations in Central European farmlands should target a diversity of habitat types needed to support a wide range of functional groups.

## Graphical abstract

We found contrasting patterns in the diversity measures of primary producers, pollinators and predators in three different habitats (road verges, crop fields and sown wildflower fields). Therefore, a diversity of habitat types is needed when multiple groups should benefit.

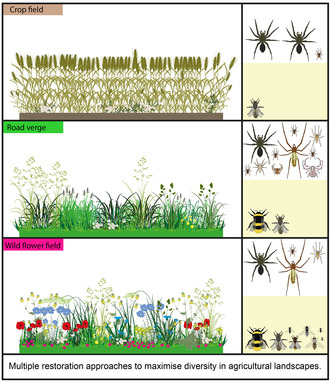

## 1 Introduction

The diversity of species and their habitats is declining globally mostly due to land‐use change caused by agriculture and consequent habitat loss (Díaz et al. 2019). About half of Europe's surface area is currently used for agriculture (European Environment Agency 2019) and is increasingly intensified (Emmerson et al. 2016). Over the last decades, that has led to the decrease in diverse semi‐natural habitats such as grassland patches, road and field verges, hedgerows and woody structures (Benton, Vickery, and Wilson 2003; Emmerson et al. 2016). Such areas are important habitats for arthropods, such as wild bees or spiders, that provide important ecosystem services in farmlands, namely pollination or predation as invertebrate pest control on crops (Kaur et al. 2019; Krimmer et al. 2019). In addition, linear landscape elements such as road verges connect the fragments of semi‐natural habitat in otherwise highly converted landscapes (Kaur et al. 2019; Batáry et al. 2021). Preserving and promoting such habitats in farmlands is crucial to sustain biodiversity and food‐providing ecosystem services (e.g., Finch 2020; Maas et al. 2021; Savage et al. 2021).

With the current UN Decade on Ecosystem Restoration, the need for additional semi‐natural habitats in farmlands has been brought to international attention (UNGA 2019). The European Union's Biodiversity Strategy and the Nature Restoration Law pave the way to promote farmland biodiversity by re‐establishing semi‐natural habitats (Hermoso et al. 2022). Particularly, semi‐natural grasslands are a prominent target of such efforts because of their high biodiversity value (Habel et al. 2013). Restored semi‐natural grasslands (e.g., overseeded grassland set‐asides, or newly created grasslands via sowing diverse wildflower seed mixtures) promote plant species richness and subsequently a diverse arthropod assemblage already shortly—although not necessarily immediately—after establishment (Hyvönen et al. 2021; Hussain et al. 2022; Dolezal, Esch, and MacDougall 2022; Bihaly et al. 2024). The success of such new semi‐natural grasslands (e.g., wildflower fields) is generally higher when sowing high‐diversity seed mixtures, although site history is an important determinant of establishment success (Kiehl et al. 2010; Török et al. 2011; Brandl et al. 2022) and plant species number may decrease over time due to natural succession (Hussain et al. 2022).

Arthropod groups differ in how they respond to the establishment of species‐diverse semi‐natural grasslands (e.g., sown wildflower fields) in farmlands. The primary producers, that is, plants are largely established because of direct seeding or/and emergence from the seed bank (Bossuyt and Honnay 2008; Kiehl et al. 2010; Török et al. 2011) and create habitat for other functional groups. Groups such as pollinators benefit directly from the flowers as food resources (Balzan, Bocci, and Moonen 2014). Other functional groups, such as predators, that are less dependent on abundant and diverse flower resources, may not be influenced directly by newly established fields, because they may find an adequate habitat, for example, in grassy road verges in the landscape (Kaur et al. 2019; Maas et al. 2021).

In this paper, we aim to compare recently established, sown wildflower fields with permanent, semi‐natural road verges and the adjacent crop fields in terms of their species numbers, individual numbers and assemblage compositions to reveal differences between primary producers (i.e., plants), pollinators (i.e., bees and hoverflies—as major pollinators in the temperate zone (IPBES 2016; Klecka et al. 2018)) and predators (i.e., spiders—as they are considered important predators (Nyffeler and Sunderland 2003)).

Similar studies were conducted in Western and northern Europe (e.g., Öckinger and Smith 2007; Li et al. 2020; Hussain et al. 2022), but largely lacking from East Central Europe, more specifically from the Pannonian region. This is an urgent issue as knowledge for each region is needed for the effective implementation of the recently adopted EU's Nature Restoration Law. We aim to contribute to closing this knowledge gap with this study.

## 2 Methods

### 2.1 Study Area and Design

**FIGURE 1:**
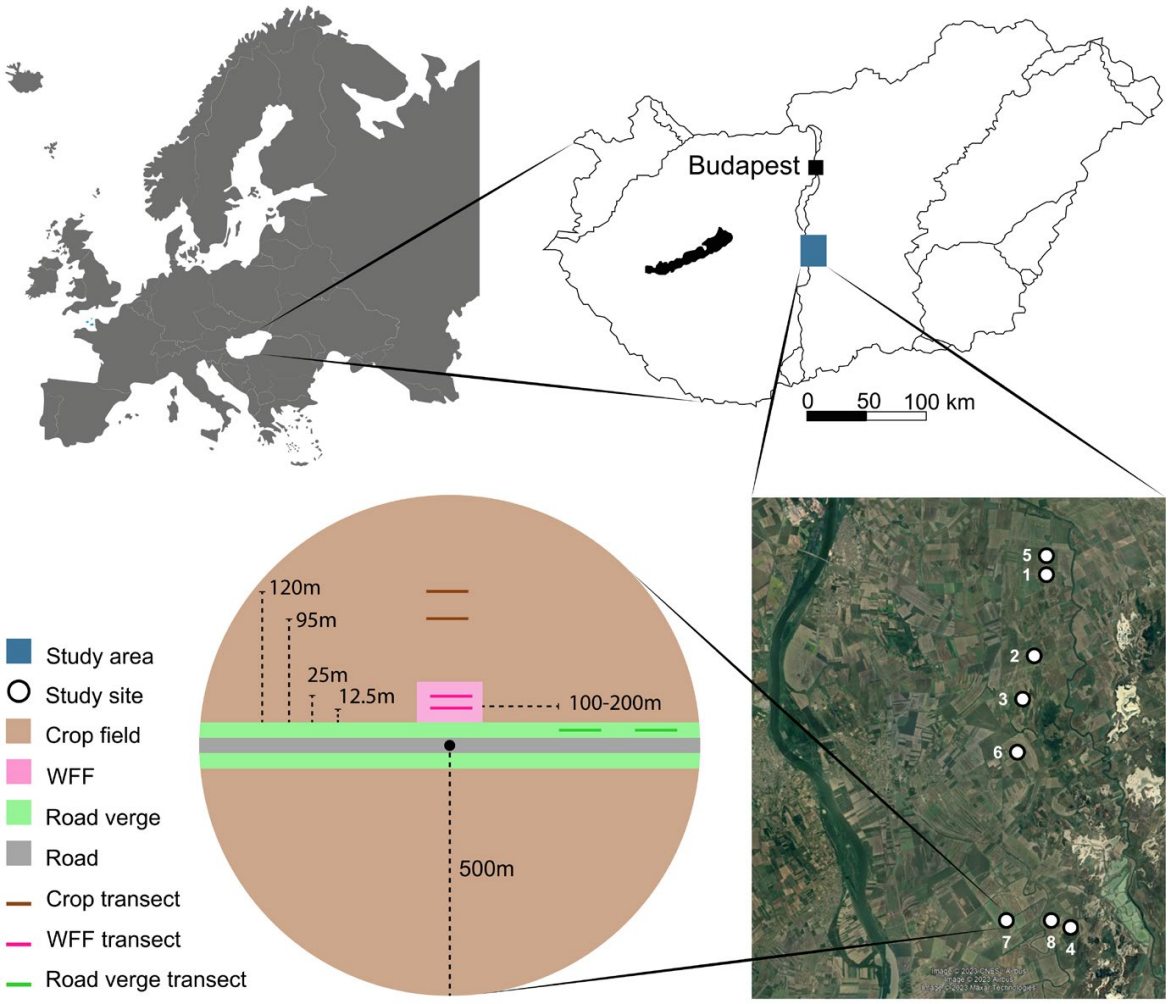
Study location within Hungary and schematic setup of the study area. The wildflower field (WFF) is set up in the middle of the circle, with two transects at 12.5 m and 25 m from the road edge. Transects in the road verge were 100–200 m from the WFF, and those in the crop fields were 95 m and 120 m from the road edge. See the locations here. Satellite image from Google maps (No date). Available at: https://www.Google.com/maps/d/edit?mid=1Aot_UHrCBp1g_BCnBW7pjCbGkojP2RA&usp=sharing (Last accessed: 20 October 2024).

We selected eight landscape circles (*r* = 500 m; Concepción et al. 2012; Grass et al. 2020) in the Great Hungarian Plain, Central Hungary (Figure 1). The landscape is characterised by vast conventionally managed (ca. 100 kg N/ha on average) agricultural fields and alternating semi‐natural grasslands and wetlands; arable land amounted to more than 40% of every landscape circle. Due to rotational agriculture, five different crops (alfalfa, barley, corn, sunflower and wheat, Table A1) were around our wildflower fields (WFFs). We pooled the crop biodiversity data irrespective of which crop was there, as it was clearly distinct from the WFF and road verges where agrochemicals and soil tillage were not applied. The road verges in the area either consist of tree hedgerows or grassy margins just a few metres wide. Each landscape circle contains a 0.5 ha‐sized (50 × 100 m) sown WFF in the middle. The fields were sown in early 2020 on the edge of former crop fields with a seed mixture of 32 local insect‐visited flowering plant species (for a detailed list of sown species, see Appendix 1 in Báldi et al. 2022) that provide food resources over the whole vegetation period and habitat for nesting, resting and mating for many animal taxa (Báldi et al. 2022).

### 2.2 Biodiversity Sampling

Within the landscape circles, we sampled the bee, hoverfly and spider assemblage and the vegetation within three habitat types, namely the WFFs, the existing road verges and inside the crop field surrounding a given WFF (Figure 1). We defined two transects in each habitat type:

1. In the WFF at 12.5 and 25 m from the crop field edge;
2. In the existing road verge at 100–200 m distance from the WFF, with the transects being in one line, separated by 25 m. The road verge was at the same crop field in which the WFF is located; and.
3. Inside the crop field at 95 and 120 m away from the crop field edge—to avoid edge effect.

We recorded vascular plant species number and cover on every transect of each habitat by using two 1 × 1 m quadrats (Martin et al. 2021) per transect (at least 10 m apart). The survey took place in June 2022, as the plant diversity of our WFFs can be estimated the most reliably during that month. In each quadrat, we recorded all vascular plant species using Király (2009), the cover of each plant species, bare soil and litter, with a total cover of 100% (i.e., no multiple vegetation layers). Species with very small cover were noted with 0.01% cover. We used the non‐crop plant species for the analyses and used species names according to The World Flora Online (http://www.worldfloraonline.org).

Bees (including wild bees and honeybees) and hoverflies were surveyed during transect walks of 50 m in length and an observation width of 1.5 m. During a net sampling time of 7.5 min (handling time of specimens excluded), we aimed to capture all observed (foraging, flying or basking) individuals (except bumblebee queens and honeybees) with a sweep net for later identification. Captured bees and hoverflies from a given transect were pooled in vials containing 70% alcohol. Specimens which were neither successfully caught nor identified to the species level in the field were noted in broad categories such as ‘wild bee’, ‘bumblebee’ or ‘hoverfly’ (22.12% of bees at the early summer sampling occasion, 23.18% of bees at the mid‐summer sampling occasion and 47.06% of hoverflies at the early summer sampling occasion). We also recorded information on the date and the location of the transect (i.e., landscape circle ID, transect ID, habitat type), measured temperature [°C] and estimated cloud cover [%] before starting the sampling to verify that the sampling was conducted during favourable weather conditions (min. temperature 18°C, average 26°C, max. 34°C, not more than 5 m/s wind and min. 0%, average 15%, max. 100% cloud cover). We sampled twice a year in 2022: early summer (late May—early June) and mid‐summer (early August). We performed the survey at a time when the target taxa are most active: in early summer between 8:30 and 17:00 and in mid‐summer between 7:00 and 17:00. In case the temperatures were higher than 34°C in mid‐summer, and the observed activity of the bees and hoverflies decreased, we suspended the work for that period (i.e., 13:00 or 14:00). Specimens were identified to the species level in the lab by Zsolt Józan and Zoltán Soltész, using Móczár (1957), Ebmer (1969, 1971), Speight (2020) and Tóth (2011) as reference guides.

Spiders were caught by suction‐sampling using a modified leaf blower (Stihl SH 86) where a gauze bag was fixed into its nozzle; the method was adapted from the BioBio project's protocol (Kovács‐Hostyánszki et al. 2013). We took five sub‐samples per transect at least 20 m apart (exceeding the length of the pollinator transect). Each of the five sub‐samples was taken within a 0.357 m internal diameter sample ring pre‐placed on the target vegetation (total sampled area per transect: 0.5 m^2^). The suction nozzle was placed over and pushed into the vegetation and moved within the sample ring for 30 s. The five sub‐samples were pooled in a polyethene zip‐seal bag and filled with 70% alcohol. Later in the lab, they were stored in a freezer until sorting. We surveyed twice a year: in landscape circles with barley and wheat 3 and 10 weeks after dandelion (*Taraxacum officinalis*) was in bloom (early May and mid‐June), and in landscape circles with corn, sunflower and alfalfa 6 and 20 weeks after dandelion bloom (late May and early September). Adult spiders were identified at the species level, while juvenile spiders (45.56% of all individuals) were identified at the family level. Heimer and Nentwig 1991 and Nentwig et al. (2023) were used as reference guides during identification.

### 2.3 Statistical Analysis

We used generalised linear mixed models (GLMM) with Poisson distribution to study the effect of the different habitat types on species and individual numbers; in the models landscape and transect IDs were nested random factors. In the case of vegetation cover, we applied beta distribution in the GLMM. We used Tukey's test to evaluate the significance of differences in species and individual/cover numbers for the three habitat groups. We included only the individuals identified to the species level for species number but included all individuals (i.e., observed‐only bees, hoverflies, and juvenile and adult spiders) to the individual number. The two sampling rounds were analysed separately for bees, while data analysis of the second sampling round was impossible for hoverflies and spiders due to the low number of individuals.

We used the glmmTMB (‘1.1.8’, Brooks et al. 2017) packages for modelling, the multcomp (‘1.4.25’, Hothorn, Bretz, and Westfall 2008) for post hoc Tukey test to know which habitat types were significantly different from each other, the tidyverse (‘2.0.0’, Wickham et al. 2019) and dplyr (‘1.1.4’, Wickham et al. 2022) packages for data manipulation and ggplot2 (‘3.5.1’, Wickham 2016) and ggpubr (‘0.6.0’, Kassambara 2023) to visualise the number of species, individuals and the vegetation cover.

We performed PERMANOVA using zero‐adjusted Bray‐Curtis dissimilarities (the number of permutations was 999) to evaluate the effect of habitat types on the assemblages of non‐crop vegetation, bees, hoverflies and spiders. We also visualised the assemblage composition of different habitat types for non‐crop vegetation, bees, hoverflies and spiders with non‐metric multidimensional scaling (NMDS). Since we did not observe individuals on every transect, a dummy species with abundance = 1 was added to all transects to facilitate the inclusion of blank or nearly empty transects (Clarke, Somerfield, and Chapman 2006; Borcard, Gillet, and Legendre 2018) and data points were jittered for better visibility. We also added convex hulls to the figures to visualise the overlap between the three habitat types. To perform the NMDS, we used the following packages: vegan (‘2.6.4’, Oksanen et al. 2022), labdsv (‘2.1.0’, Roberts 2023), TeachingDemos (‘2.13’, Snow 2024) and goeveg (‘0.7.5’, Goral and Schellenberg 2024).

Based on the average species composition in each taxon in each habitat type, we calculated the Bray–Curtis dissimilarity (as a beta diversity index; see Schroeder and Jenkins 2018) for pairwise comparisons of habitat types (road verge—crop; WFF—crop; WFF—road verge) for non‐crop vegetation, bees, hoverflies and spiders. Finally, we calculated the significance of difference in dissimilarity using the adonis function of the vegan package (‘2.6.4’, Oksanen et al. 2022).

We used the R statistical environment (version 4.4.0) to perform all the analyses.

## 3 Results

### 3.1 Primary Producers—Plants

**FIGURE 2:**
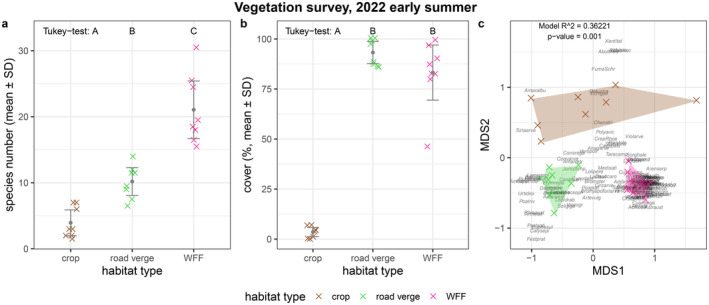
Results of the non‐crop plant (a) species number, (b) cover percentages and (c) assemblage composition. The colourful crosses (×) in (a) and (b) show the species number and vegetation cover in each of the eight landscape plots separated by habitat types (mean ± SD); full circles crosses are jittered to increase visibility. Letters A, B and C above the boxplots in subplots (a) and (b) indicate significant differences according to the Tukey test. In (c), the observed species are abbreviated with the first four letters of their genus name and the first four letters of their species name—a list of species names and their abbreviations can be found in the Appendix Dataset; sampling sites are represented by colourful crosses (×); species names and crosses are jittered for better visibility. *R* ^2^ and *p*‐value of the PERMANOVA and stress value of the NMDS are indicated in the top right corner. For better visibility, the NMDS subplot can be found in the Appendix in a separated, enlarged form (Figure A1).

**FIGURE A1:**
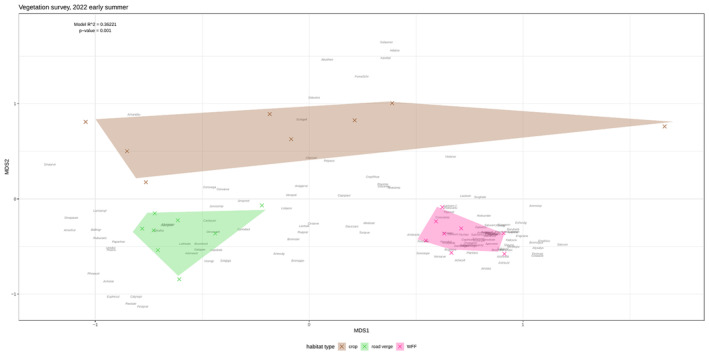
Results of the non‐crop plant assemblage composition. The observed species are abbreviated with the first four letters of their genus name and the first four letters of their species name – a list of species names and their abbreviations can be found in Appendix Dataset S1; sampling sites are represented by colourful crosses (×); species names and crosses are jittered for better visibility. *R* ^2^ and *p*‐value of the PERMANOVA and stress value of the NMDS are indicated in the top right corner.

The number of non‐crop plant species was significantly different in the three habitats according to the GLMM (Tukey tests), with the crop field habitats having the lowest species numbers, followed by road verges and WFFs (all *p* < 0.001; Figure 2a; Table 1; Table A2). We found similar results for the cover percentages of non‐crop plant species: the crop habitats were covered with only a few percent of non‐cultivated plants, while in the WFF and the road verge habitats, the cover was significantly higher (all *p* < 0.001; Figure 2b; Table 1; Table A2). The NMDS analysis showed that the assemblage composition of the three habitats is distinctly different (PERMANOVA: *R* ^2^ = 0.36221, *p* = 0.001; Figure 2c; Figure A1, Table A3). According to the Bray–Curtis dissimilarity, WFF and crop habitats were the most different in their plant assemblages, while WFF and road verge habitats were more similar (Table 2).

**TABLE 1:** Summary statistics of the generalised linear mixed models (GLMMs) evaluating the relationship between species or individual numbers or cover and different habitat types (semi‐natural road verge, crop field, sown wildflower field [WFF]) for non‐crop vegetation, bees, hoverflies and spiders at the early and mid‐summer sampling occasions. Significant *p*‐values are in bold.

	Explanatory variables	Estimate	SE	*Z*	*p*
Vegetation, early summer species number	Intercept (crop)	1.4028	0.1280	10.9564	**< 0.0001**
	Road verge	0.9183	0.1501	6.1184	**< 0.0001**
	WFF	1.6447	0.1391	11.8200	**< 0.0001**
Vegetation, early summer cover	Intercept (crop)	−3.6572	0.4093	−8.9360	**< 0.0001**
	Road verge	6.1479	0.5578	11.0218	**< 0.0001**
	WFF	5.4526	0.5216	10.4547	**< 0.0001**
Bees, early summer species number	Intercept (crop)	< 0.0001	1.0000	< 0.0001	1.0000
	Road verge	0.4055	1.0541	0.3847	0.7005
	WFF	1.0986	1.0104	1.0873	0.2769
Bees, early summer individual number	Intercept (crop)	−1.0576	0.4900	−2.1582	**0.0309**
	Road verge	1.5053	0.5117	2.9420	**0.0033**
	WFF	3.2198	0.4783	6.7312	**< 0.0001**
Bees, mid‐summer species number	Intercept (crop)	0.2877	0.5000	0.5754	0.5650
	Road verge	0.3409	0.5627	0.6058	0.5446
	WFF	0.6286	0.5244	1.1987	0.2306
Bees, mid‐summer individual number	Intercept (crop)	−1.3430	0.4927	−2.7255	**0.0064**
	Road verge	2.1946	0.5623	3.9027	**0.0001**
	WFF	3.3140	0.5446	6.0852	**< 0.0001**
Hoverflies, early summer species number	Intercept (road verge)	< 0.0001	0.5000	< 0.0001	1.0000
	WFF	0.2624	0.5718	0.4589	0.6463
Hoverflies, early summer individual number	Intercept (crop)	−1.1186	0.5107	−2.1902	**0.0285**
	Road verge	1.1241	0.6054	1.8568	0.0633
	WFF	1.8438	0.5495	3.3552	**0.0008**
Spiders, early summer species number	Intercept (crop)	0.2346	0.6101	0.384	0.701
	Road verge	0.7714	0.6063	1.272	0.203
	WFF	−0.1279	1.3108	−0.098	0.922
Spiders, early summer individual number	Intercept (crop)	−1.2687	0.5599	−2.2661	**0.0234**
	Road verge	2.2185	0.5781	3.8379	**0.0001**
	WFF	0.0513	0.6642	0.0772	0.9385

**TABLE 2:** Bray‐Curtis dissimilarities of pairwise comparisons of non‐crop vegetation, bees, hoverflies, and spiders in three habitat types (semi‐natural road verge, crop field, sown wildflower field [WFF]) in an agricultural landscape. A higher value indicates a bigger dissimilarity, that is, a bigger difference between two habitats. Values can be between 0 (no difference) and 1 (complete difference).

	Pairwise comparison	Bray–Curtis dissimilarity
Vegetation, early summer	road verge – crop	0.7152
	WFF – crop	0.8069
	WFF—road verge	0.7417
Bees, early summer	road verge—crop	0.3043
	WFF—crop	0.8298
	WFF—road verge	0.8000
Bees, mid‐summer	road verge—crop	0.4839
	WFF—crop	0.7946
	WFF – road verge	0.7488
Hoverflies, early summer	WFF – road verge	0.3200
Spiders, early summer	road verge – crop	0.5556
	WFF – crop	0.1500
	WFF – road verge	0.5152

### 3.2 Pollinators—Wild and Honey Bees and Hoverflies

**FIGURE 3:**
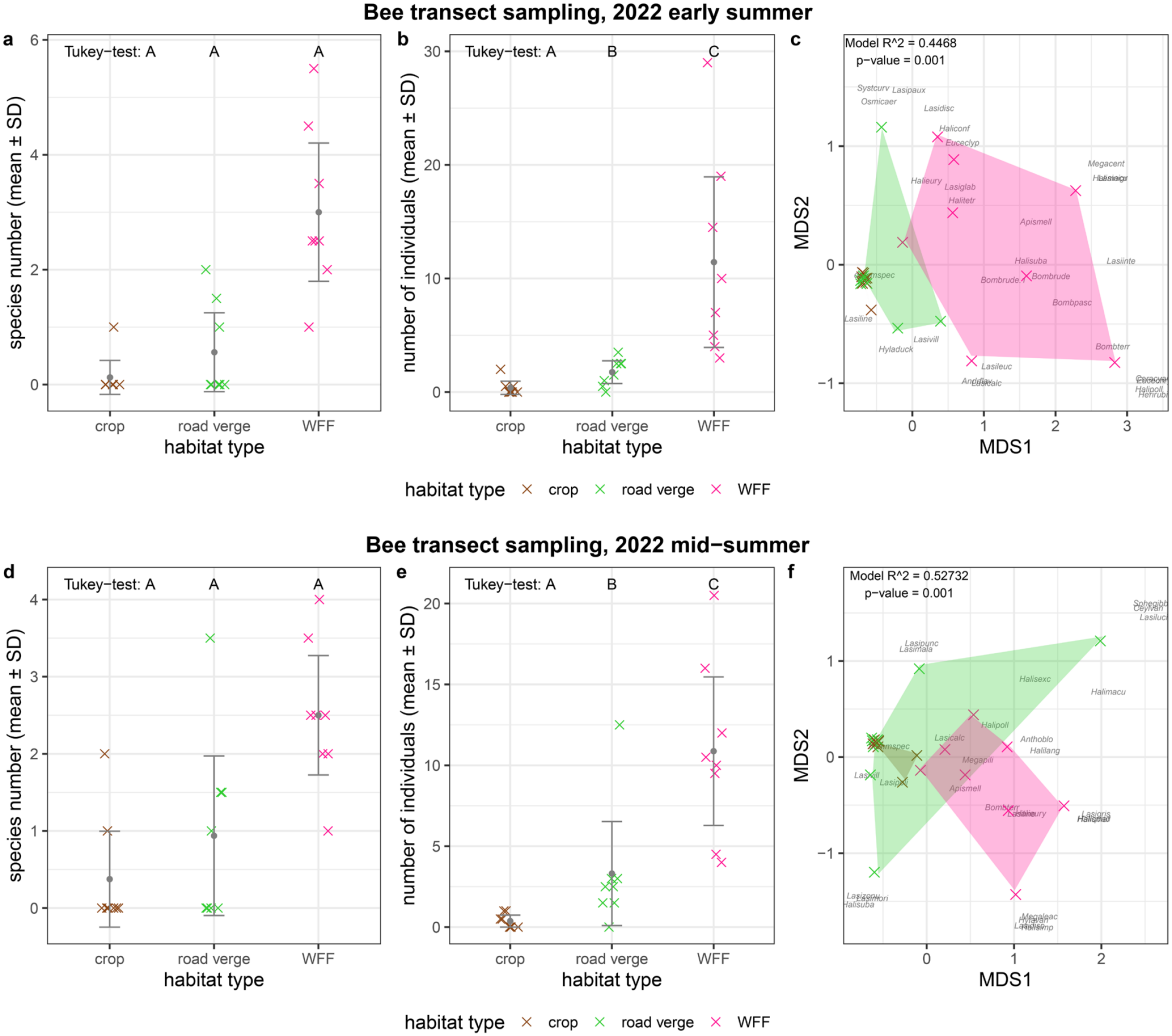
Results of the bee (a) species number, (b) individual number, (c) assemblage composition in early summer and mid‐summer (d, e, f), respectively. The crosses (×) in (a) and (b) show the number of species and individuals in each of the eight landscape plots separated by habitat types (mean ± SD); crosses are jittered to increase visibility. Letters A, B and C above the boxplots in subplots (a) and (b) indicate significant differences according to the Tukey test. In (c), the observed species are noted by the first four letters of their genus name and the first four letters of their species name—a list of species names and their abbreviations can be found in Appendix Dataset; sampling sites are represented by colourful crosses (×); species names and crosses are jittered for better visibility. *R* ^2^ and *p*‐value of the PERMANOVA and stress value of the NMDS are indicated in the top right corner. Note that only individuals identified at the species level were included in the species number and NMDS indices, while all observed individuals were included in the abundance indices.

We found no significant differences in species numbers of bees (Figure 3a,d; Table 1; Table A2). We observed significant differences between the three habitats' individual numbers, with only a few bee individuals inside the crop fields, intermediate levels in the road verge habitat and highest in the WFF (Figure 3b,e; Table 1; Table A2). In the early summer sampling occasion, road verge and WFF habitats were different in their bee assemblage composition according to the NMDS (*R* ^2^ = 0.4468, *p*‐value = 0.001; Figure 3c; Table A3). In the later sampling session, the assemblages of the bees captured within the crop field overlapped with the assemblage of the road verge, and the assemblage of the road verge and WFF habitats partially overlapped with each other, but still differed significantly (*R* ^2^ = 0.52732, *p*‐value = 0.001; Figure 3f; Table A3). In terms of species compositions, the pairwise Bray–Curtis dissimilarities suggest a bigger difference between WFF and crop, and WFF and road‐verges, while showing a smaller dissimilarity between crop and road verge in both sampling occasions (Table 2).

**FIGURE 4:**
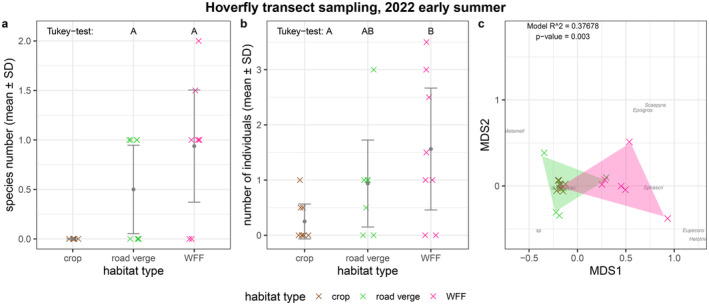
Results of the hoverfly (a) species number, (b) individual number, (c) assemblage composition in early summer. The colourful crosses (×) in (a) and (b) show the number of species and individuals in each of the eight landscape plots separated by habitat types (mean ± SD); crosses are jittered to increase visibility. Letters A and B above the boxplots in subplots (a) and (b) indicate significant differences according to the Tukey test. In (c), the observed species are noted by the first four letters of their genus name and the first four letters of their species name—a list of species names and their abbreviations can be found in Appendix Dataset; sampling sites are represented by colourful crosses (×); species names and crosses are jittered for better visibility. *R* ^2^ and *p*‐value of the PERMANOVA and stress value of the NMDS are indicated in the top right corner. Note that only individuals identified at the species level were included in the species number and NMDS indices, while all observed individuals were included in the abundance indices.

In early summer, we did not capture any hoverflies that could be identified at the species level in the crop habitats and only a few species were captured on the transects in the road verge and WFF habitats. We did not find any significant differences in either species (Figure 4a; Table 1; Table A2). Hoverfly individual numbers at the WFF habitats were significantly higher compared to the crop habitats (Figure 4b; Table 1; Table A2). The NMDS plot showed some overlap between the road verge and the WFF habitats‘hoverfly assemblage composition at the earlier sampling occasion (PERMANOVA: *R* ^2^ = 0.37678, *p* = 0.003; Figure 4c, Table A3). In terms of species compositions, the road verges and the WFF habitats were similar, as the pairwise Bray–Curtis dissimilarity suggests (Table 2). The number of species and individuals captured and observed during the second sampling round was too low to use in a robust statistical analysis and to have a reliable conclusion.

### 3.3 Predators—Spiders

**FIGURE 5:**
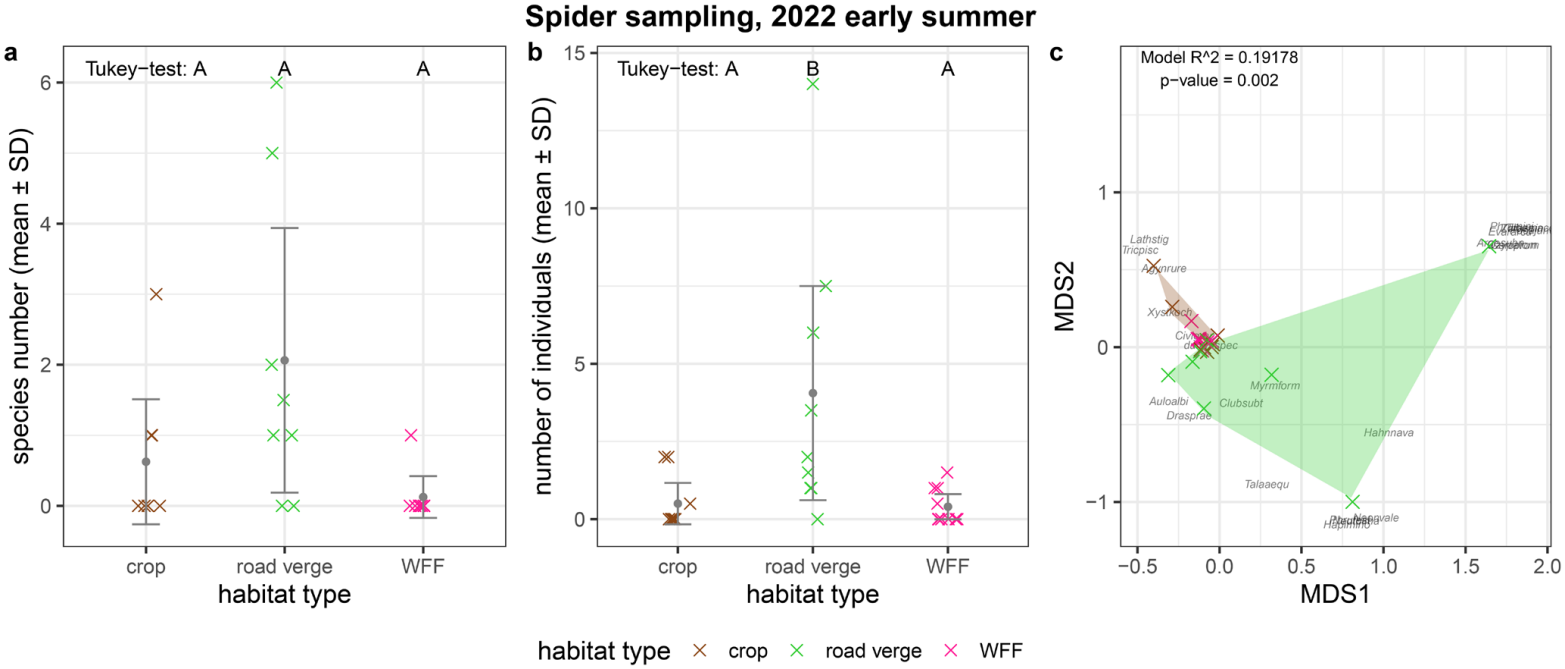
Results of the spider (a) species number, (b) individual number, (c) assemblage composition in early summer. The colourful crosses (×) in (a) and (b) show the number of species and individuals in each of the eight landscape plots separated by habitat types (mean ± SD); crosses are jittered to increase visibility. Letters A and B above the boxplots in subplots (a) and (b) indicate significant differences according to the Tukey test. In (c), the observed species are noted by the first four letters of their genus name and the first four letters of their species name—a list of species names and their abbreviations can be found in Appendix Dataset; sampling sites are represented by colourful crosses (×); species names and crosses are jittered for better visibility. *R* ^2^ and *p*‐value of the PERMANOVA, and stress value of the NMDS are indicated in the top right corner. Note that only individuals identified at the species level were included in the species number and NMDS indices, while all observed individuals were included in the abundance indices.

According to the Tukey test, the number of spider species was similar in the three habitats (Figure 5a; Tables 1 and 2). However, we found a significantly lower number of individuals (Figure 5b; Table 1; Table A2) at the crop and the WFF habitats compared to the road verges in the early summer sampling. This could also be observed in the spider assemblage composition, where the crop and WFF differed from the road verge (*R* ^2^ = 0.19178, *p* = 0.002; Figure 5c; Table A3). In terms of species compositions, the pairwise Bray‐Curtis dissimilarities suggest a bigger difference between road verge and crop, and WFF and road verge, while showing a smaller dissimilarity between crop and WFF (Table 2). Due to the high number of juvenile individuals that could not be identified at the species level, the data from the second sampling round was omitted.

## 4 Discussion

In this study, we aimed to compare different habitat types regarding their functional group composition in farmlands. We collected data during 1 year, once for vegetation during peak flowering time and twice for arthropods, which raises the uncertainty resulting from large annual variability of arthropod populations. We acknowledge the limitation of a 1 year study, but as we found evidence that the communities inhabiting crop fields, road verges or WFFs are distinguished from each other, our conclusions can be regarded as valid. We can offer a snapshot into the restoration process 2 years after the establishment of the WFFs that turned a species‐poor arable field into a new grassland rich in forbs. We also recognise the issue of botanical sampling, as vegetation data is based on rather few 1 × 1 m quadrats per landscape circle. However, the observed pattern of plants is robust, and fits to field observations, thus is an appropriate method to predict plant and related insect assemblages in future restoration efforts. According to our results, crop fields are not suitable habitats for the investigated functional groups because they do not find their required resources and nesting places; however crop fields heavily depend on the ecosystem services of these functional groups, thus the existing or newly created surrounding semi‐natural habitats (i.e., road verge, WFF) are important parts of the agricultural landscapes. The combination of these habitats is important since the demands of different functional groups are met in dissimilar ways: we found that pollinators (bees and hoverflies) and predators (spiders) did not favour the same habitat types. While pollinators preferred the flower‐rich wildflower fields, predators were more abundant in the densely vegetated road verges. We also found that the different habitats had mostly non‐overlapping species composition (especially non‐crop vegetation, bees in the early summer sampling occasion and spiders), suggesting fine spatial scale differences within the taxa.

### 4.1 Primary Producers—Plants

Unsurprisingly, the crop fields were the most species‐poor habitat type in terms of plant species due to the frequent soil ploughing, the subsequent crop rotation and herbicide treatments. Non‐crop plant species are regularly eradicated in intensive agriculture as they may host crop pests (Kumar, Bhowmick, and Ray 2021) and are considered weeds potentially interfering with crop growth (Gallandt and Weiner 2015; Korav et al. 2018). Road verges are valuable semi‐natural habitats in farmland landscapes due to their connectivity function (Phillips et al. 2020; Dániel‐Ferreira et al. 2022) and important abiding habitat in an otherwise impoverished arable surrounding (Phillips et al. 2020). In our study, we found these road verges to mainly consist of a few plant species, such as grasses and weedy, generalist and ruderal species, with few flower resources. That is in strong contrast to the sown WFFs, where we found more *specialists* (according to the definition of Borhidi (1995) and Horváth et al. (1995)), including sown plant species, but even more species that had emerged from the soil seed bank or dispersed into the fields (Malo and Suárez 1995). Continued management, such as mowing, will be needed for a sustained diverse habitat (Báldi, Batáry, and Kleijn 2013; Kiss et al. 2017). The number of plant species in the WFF habitat was the highest and may therefore support more diverse and complex insect assemblages (Ebeling et al. 2018). The three habitat types had distinct plant assemblages that supported separate pollinator and predator assemblages, a finding which concurs with previous studies (Schaffers et al. 2008; Hussain et al. 2021; Brandl et al. 2022).

Species associated with the crop habitat were mostly *weeds* (classification after Borhidi (1995)), while the road verges mainly hosted *disturbance tolerants*. The WFFs were characterised by *natural pioneers* and *stress tolerants* for the seeded and naturally appearing species.

### 4.2 Pollinators—Wild and Honey Bees and Hoverflies

We observed rather few bees and hoverflies inside the crop field, although some species of the latter lay eggs close to aphid colonies (Almohamad et al. 2007; Almohamad, Verheggen, and Haubruge 2009; Miličić et al. 2021; Vujanović et al. 2023) and are considered an important pest control (and pollinator) agent (Almohamad et al. 2007; Rodríguez‐Gasol et al. 2020). Since most crops in our study area were wind‐pollinated, it might not surprise to observe only a few pollinators, although honey bees have been shown to forage on maize pollen, among others (Danner, Härtel, and Steffan‐Dewenter 2014). In the road verges, bees were significantly less abundant than in the WFFs, which can be explained by the wide array of pollinator‐friendly plant species in the latter and the low number of herbs in the verges (Albrecht et al. 2020; Threadgill et al. 2020; Wen et al. 2022). A further reason might be the higher percentage of bare soil in the WFFs, which ground‐nesting bees may profit from for nesting (Gardein et al. 2022). We observed a similar pattern for hoverfly species numbers, although individual numbers did not significantly differ between road verges and WFFs. This observation is supposedly due to a preference for areas with higher plant density (Dániel‐Ferreira et al. 2022) and shelter from predators or—for larvae—the provision of additional food resources in road verges (Sutherland, Sullivan, and Poppy 2001). Therefore, we conclude that the pollinator groups do not benefit equally from newly established wildflower fields, but for hoverflies, permanent semi‐natural grasslands such as road verges may be more important. Pairwise Bray–Curtis dissimilarities were the lowest among crops and road verges, indicating higher similarity in terms of their bee assemblages (Table 2).

### 4.3 Predators—Spiders

Although spiders are considered important pest control agents in farmland (Nyffeler and Sunderland 2003; Michalko et al. 2019), we found only a few spiders in the crop fields (e.g., *Agyneta rurestris, Trichoncoides piscator, Xysticus kochi*). This may be due to regular ploughing that recurrently erases the spider populations (and their prey) in the crop fields (Schneider, Krauss, and Steffan‐Dewenter 2013; Plath et al. 2021), meaning that the road verges could become refugia in the absence of more natural habitat. The varied and dense vegetation structure provided by the road verge habitat can be an important benefit for spiders (Balzan, Bocci, and Moonen 2014; Plath et al. 2021; Mei et al. 2021). Surprisingly, the WFFs hosted only a few spiders, supposedly because the time since the establishment of the field was insufficient for them to occupy the new habitat (Maas et al. 2021; Hussain et al. 2021) or because the vegetation is not yet dense enough to provide optimal habitat (McDonald 2007). WFFs have the potential to provide additional resources to predators, such as undisturbed overwintering and reproductional sites and increased prey resources (Hoffmann et al. 2021; Mei et al. 2021; Plath et al. 2021), but some of these are also provided by road verges. Furthermore, sown WFFs enhance pest control in the adjacent or surrounding agriculturally managed fields, as not only spiders but other pest control agent groups are also promoted (Blaauw and Isaacs 2012; Albrecht et al. 2020; Bischoff et al. 2022; but see Török et al. 2021).

We found generalist grassland spider species in the road verge habitats (e.g., *Aulonia albimana* ; *Talavera aequipes* ) and disturbance tolerant agrobiont species (i.e., that reach high dominance in agroecosystems; Samu and Szinetár 2002) inside the crop fields (e.g., *Agyneta rurestris*). The latter use road verges as overwintering sites when the crop fields lay bare. Our WFFs may be similar to the crop field in that they are relatively newly established and do not have a complex vertical structure yet. We expect that spiders will increasingly use the WFFs habitats within a few years because the WFFs will become more similar to the road verges—currently, their assemblage composition is already moderately similar (Table 2)—as grass species will colonise the WFFs and more vertical structures will develop.

### 4.4 The Broader Context

Our results suggest no habitat fits all, and a diversity of habitat types is key if different functional groups are to be supported (Balzan, Bocci, and Moonen 2014; Gayer et al. 2021; Hussain et al. 2022). Furthermore, our results show similar patterns for the studied insect groups as in other agricultural systems in western and northern Europe. In conclusion and beyond our results, there is massive evidence of the beneficial role of non‐crop habitats in supporting a wide range of essential ecosystem service providers and biodiversity (e.g., Haaland, Naisbit, and Bersier 2011; Balzan, Bocci, and Moonen 2014; Dolezal, Esch, and MacDougall 2022). Our study showed, however, that different non‐crop habitats support different functional groups; thus the ecology of the different targeted functional groups should be taken into account when planning effective conservation and restoration (Holl and Aide 2010). Beyond diversity protection per se, the maintenance of ecosystem functioning also requires a diversity of habitats on the landscape. Such knowledge will be valuable input for the implementation of the EU's Nature Restoration Law by member states when agricultural landscapes and pollinators are targeted, and more broadly to the transformation of agriculture (Báldi et al. 2023). Note, however, that due to large yearly variations in arthropod abundance, and the diversity of farm systems across Europe, these results need to be validated locally before applying to the design of local or regional multifunctional landscapes. Thus, we encourage further research to help fill the knowledge gaps, especially in East Central European landscapes.

## Author Contributions

**Flóra Vajna:** data curation (equal), formal analysis (lead), visualization (lead), writing – original draft (lead), writing – review and editing (equal). **Raoul Pellaton:** data curation (equal), writing – original draft (supporting), writing – review and editing (equal). **Csaba Molnár:** data curation (equal), writing – review and editing (equal). **Zoltán Soltész:** formal analysis (equal), writing – review and editing (equal). **Nikolett Gallé‐Szpisjak:** formal analysis (equal), visualization (equal), writing – review and editing (equal). **Áron Domonkos Bihaly:** data curation (equal), writing – review and editing (equal). **András Báldi:** conceptualization (lead), funding acquisition (lead), writing – original draft (supporting), writing – review and editing (equal).

## Conflicts of Interest

The authors declare no conflicts of interest.

## Supporting information


Data S1.

## Data Availability

Data are available in the article Appendix S1.

## Appendix A

**TABLE A1:** List of crop species cultivated at different fields, with their size in hectares.

Field number	Crop species	Field size (ha)
1	Corn	157.55
2	Barley	60.75
3	Wheat	50.68
4	Alfalfa	50.52
5	Corn	232.97
6	Barley	108.14
7	Barley	55.97
8	Sunflower	72.02

**TABLE A2:** Summary statistics of the Tukey test evaluating the relationship between species or individual number or cover and different habitat types (semi‐natural road verge, crop field, sown wildflower field [WFF]) for non‐crop vegetation, bees, hoverflies and spiders at the early and mid‐summer sampling occasions. Significant *p*‐values are in bold.

	Estimate	SE	*Z*	*p*
Vegetation, early summer species number				
Road verge – crop	0.9183	0.1501	6.1180	**< 1e‐04**
WFF – crop	1.6447	0.1391	11.8200	**< 1e‐09**
WFF – road verge	0.7263	0.0954	7.6130	**< 1e‐09**
Vegetation, early summer cover				
Road verge – crop	6.1479	0.5578	11.0220	**< 1e‐04**
WFF – crop	5.4526	0.5216	10.4550	**< 1e‐04**
WFF – road verge	−0.6952	0.4045	−1.7190	0.1950
Bees, early summer species number				
Road verge – crop	0.4055	1.0541	0.3850	0.9160
WFF – crop	1.0986	1.0104	1.0870	0.5000
WFF – road verge	0.6931	0.3632	1.9080	0.1230
Bees, early summer individual number				
Road verge – crop	1.5053	0.5117	2.9420	**0.0086**
WFF – crop	3.2198	0.4783	6.7310	**< 1e‐04**
WFF – road verge	1.7145	0.2899	5.9140	**< 1e‐04**
Bees, mid‐summer species number				
Road verge – crop	0.3409	0.5627	0.6060	0.8110
WFF – crop	0.6286	0.5244	1.1990	0.4420
WFF – road verge	0.2877	0.3028	0.9500	0.5970
Bees, mid‐summer individual number				
Road verge – crop	2.1946	0.5623	3.9030	**0.0003**
WFF – crop	3.3140	0.5446	6.0850	**< 1e‐04**
WFF – road verge	1.1194	0.3795	2.9500	**0.0086**
Hoverflies, early summer species number				
WFF – road verge	0.2624	0.5718	0.4590	0.6460
Hoverflies, early summer individual number				
Road verge – crop	1.1241	0.6054	1.8570	0.1474
WFF – crop	1.8438	0.5495	3.3550	**0.0022**
WFF – road verge	0.7197	0.4093	1.7580	0.1793
Spiders, early summer species number				
Road verge – crop	0.7714	0.6063	1.2720	0.3840
WFF – crop	−0.1279	1.3108	−0.0980	0.9940
WFF – road verge	−0.8992	1.0845	−0.8290	0.6620
Spiders, early summer individual number				
Road verge – crop	2.2185	0.5781	3.8380	**0.0004**
WFF – crop	0.0513	0.6642	0.0770	0.9967
WFF – road verge	−2.1672	0.5626	−3.8520	**0.0003**

**TABLE A3:** NMDS model results from analysing the assemblage composition for non‐crop vegetation, bees, hoverflies and spiders at the early and mid‐summer sampling occasions. Significant *p*‐values are in bold.

	df	Sum of squares	*R* ^2^	*F*	*p*
Vegetation, early summer					
Model	2	2.9642	0.3622	5.963	**0.001**
Residual	21	5.2195	0.6378		
Total	23	8.1836	1.0000		
Bees, early summer					
Model	2	1.8080	0.4468	8.480	**0.001**
Residual	21	2.2387	0.5532		
Total	23	4.0467	1.0000		
Bees, mid‐summer					
Model	2	2.3723	0.5273	11.714	**0.001**
Residual	21	2.1265	0.4727		
Total	23	4.4987	1.0000		
Hoverflies, early summer					
Model	2	0.2972	0.3768	6.348	**0.003**
Residual	21	0.4915	0.6232		
Total	23	0.7887	1.0000		
Spiders, early summer					
Model	2	0.3630	0.1918	2.491	**0.002**
Residual	21	1.5297	0.8082		
Total	23	1.8927	1.0000		

## References

[ece370711-bib-0001] Albrecht, M. , D. Kleijn , N. M. Williams , et al. 2020. “The Effectiveness of Flower Strips and Hedgerows on Pest Control, Pollination Services and Crop Yield: A Quantitative Synthesis.” Ecology Letters 23, no. 10: 1488–1498. 10.1111/ele.13576. 32808477 PMC7540530

[ece370711-bib-0002] Almohamad, R. , F. J. Verheggen , F. Francis , and É. Haubruge . 2007. “Predatory Hoverflies Select Their Oviposition Site According to Aphid Host Plant and Aphid Species.” Entomologia Experimentalis et Applicata 125, no. 1: 13–21. 10.1111/j.1570-7458.2007.00596.x.

[ece370711-bib-0003] Almohamad, R. , F. J. Verheggen , and É. Haubruge . 2009. “Searching and Oviposition Behavior of *Aphidophagous hoverflies* (Diptera: Syrphidae): A Review.” Biotechnology, Agronomy, Society and Environment 13, no. 3: 467–481.

[ece370711-bib-0004] Báldi, A. , P. Batáry , and D. Kleijn . 2013. “Effects of Grazing and Biogeographic Regions on Grassland Biodiversity in Hungary – Analysing Assemblages of 1200 Species.” Agriculture, Ecosystems & Environment 166: 28–34. 10.1016/j.agee.2012.03.005.

[ece370711-bib-0005] Báldi, A. , K. Öllerer , A. Wijkman , G. Brunori , A. Máté , and P. Batáry . 2023. “Roadmap for Transformative Agriculture: From Research Through Policy Towards a Liveable Future in Europe.” In Advances in Ecological Research, 131–154. Amsterdam, Netherlands: Elsevier. 10.1016/bs.aecr.2023.09.007.

[ece370711-bib-0006] Báldi, A. , R. Pellaton , Á. D. Bihaly , et al. 2022. “Improving Ecosystem Services in Farmlands: Beginning of a Long‐Term Ecological Study With Restored Flower‐Rich Grasslands.” Ecosystem Health and Sustainability 8, no. 1: 2090449. 10.1080/20964129.2022.2090449.

[ece370711-bib-0007] Balzan, M. V. , G. Bocci , and A. C. Moonen . 2014. “Augmenting Flower Trait Diversity in Wildflower Strips to Optimise the Conservation of Arthropod Functional Groups for Multiple Agroecosystem Services.” Journal of Insect Conservation 18, no. 4: 713–728. 10.1007/s10841-014-9680-2.

[ece370711-bib-0008] Batáry, P. , V. Rösch , C. F. Dormann , and T. Tscharntke . 2021. “Increasing Connectivity Enhances Habitat Specialists but Simplifies Plant‐Insect Food Webs.” Oecologia 195, no. 2: 539–546. 10.1007/s00442-020-04830-6. 33367959 PMC7882472

[ece370711-bib-0009] Benton, T. G. , J. A. Vickery , and J. D. Wilson . 2003. “Farmland Biodiversity: Is Habitat Heterogeneity the Key?” In Trends in Ecology and Evolution 18, no. 4: 182–188. 10.1016/S0169-5347(03)00011-9.

[ece370711-bib-0010] Bihaly, Á. D. , I. S. Piross , R. Pellaton , et al. 2024. “Landscape‐Wide Floral Resource Deficit Enhances the Importance of Diverse Wildflower Plantings for Pollinators in Farmlands.” Agriculture, Ecosystems and Environment 367: 108984. 10.1016/j.agee.2024.108984.

[ece370711-bib-0011] Bischoff, A. , A. Pollier , Y. Tricault , et al. 2022. “A Multi‐Site Experiment to Test Biocontrol Effects of Wildflower Strips in Different French Climate Zones.” Basic and Applied Ecology 62: 33–44. 10.1016/j.baae.2022.04.003.

[ece370711-bib-0012] Blaauw, B. R. , and R. Isaacs . 2012. “Larger Wildflower Plantings Increase Natural Enemy Density, Diversity, and Biological Control of Sentinel Prey, Without Increasing Herbivore Density.” Ecological Entomology 37, no. 5: 386–394. 10.1111/j.1365-2311.2012.01376.x.

[ece370711-bib-0013] Borcard, D. , F. Gillet , and P. Legendre . 2018. Numerical Ecology With R. 2nd ed. New York: Springer.

[ece370711-bib-0014] Borhidi, A. L. 1995. “Social Behaviour Types, the Naturalness and Relative Indicator Values of the Higher Plants in the Hungarian Flora.” Acta Botanica Hungarica 1–2, no. 39: 97–188.

[ece370711-bib-0015] Bossuyt, B. , and O. Honnay . 2008. “Can the Seed Bank Be Used for Ecological Restoration? An Overview of Seed Bank Characteristics in European Communities.” Journal of Vegetation Science 19, no. 6: 875–884. 10.3170/2008-8-18462.

[ece370711-bib-0016] Brandl, M. , R. I. Hussain , B. Maas , et al. 2022. “Improving Insect Conservation Values of Agri‐Environment Schemes Through Diversified Seed Mixtures.” Biological Conservation 269: 109530. 10.1016/j.biocon.2022.109530.

[ece370711-bib-0017] Brooks, M. E. , K. Kristensen , K. J. van Benthem , et al. 2017. “glmmTMB Balances Speed and Flexibility Among Packages for Zero‐Inflated Generalized Linear Mixed Modeling.” R Journal 9, no. 2: 378–400. 10.32614/RJ-2017-066.

[ece370711-bib-0018] Clarke, K. R. , P. J. Somerfield , and M. G. Chapman . 2006. “On Resemblance Measures for Ecological Studies, Including Taxonomic Dissimilarities and a Zero‐Adjusted Bray‐Curtis Coefficient for Denuded Assemblages.” Journal of Experimental Marine Biology and Ecology 330, no. 1: 55–80. 10.1016/j.jembe.2005.12.017.

[ece370711-bib-0019] Concepción, E. D. , M. Díaz , D. Kleijn , et al. 2012. “Interactive Effects of Landscape Context Constrain the Effectiveness of Local Agri‐Environmental Management.” Journal of Applied Ecology 49: 695–705. 10.1111/j.1365-2664.2012.02131.x.

[ece370711-bib-0020] Dániel‐Ferreira, J. , Å. Berggren , J. Wissman , and E. Öckinger . 2022. “Road Verges Are Corridors and Roads Barriers for the Movement of Flower‐Visiting Insects.” Ecography 2: 1–11. 10.1111/ecog.05847.

[ece370711-bib-0021] Danner, N. , S. Härtel , and I. Steffan‐Dewenter . 2014. “Maize Pollen Foraging by Honey Bees in Relation to Crop Area and Landscape Context.” Basic and Applied Ecology 15, no. 8: 677–684. 10.1016/j.baae.2014.08.010.

[ece370711-bib-0022] Díaz, S. , J. Settele , E. S. Brondízio , et al. 2019. “Pervasive Human‐Driven Decline of Life on Earth Points to the Need for Transformative Change.” Science 366: eaax3100. 10.1126/science.aaw3100. 31831642

[ece370711-bib-0023] Dolezal, A. J. , E. H. Esch , and A. S. MacDougall . 2022. “Restored Marginal Farmland Benefits Arthropod Diversity at Multiple Scales.” Restoration Ecology 30, no. 1: 13485. 10.1111/rec.13485.

[ece370711-bib-0024] Ebeling, A. , J. Hines , L. R. Hertzog , et al. 2018. “Plant Diversity Effects on Arthropods and Arthropod‐Dependent Ecosystem Functions in a Biodiversity Experiment.” Basic and Applied Ecology 26: 50–63. 10.1016/j.baae.2017.09.014.

[ece370711-bib-0025] Ebmer, P. A. W. 1969. “Die Bienen des Genus Halictus Latr. S. L. im Grossraum von Linz (Hymenoptera, Apidae), Teil I.” Naturkundliches Jahrbuch der Stadt Linz 1969: 133–183.

[ece370711-bib-0026] Ebmer, P. A. W. 1971. “Die Bienen des Genus Halictus Latr. S. L. im Grossraum von Linz (Hymenoptera, Apidae), Teil III.” Naturkundliches Jahrbuch der Stadt Linz 1971: 63–156.

[ece370711-bib-0027] Emmerson, M. , M. B. Morales , J. J. Oñate , et al. 2016. “How Agricultural Intensification Affects Biodiversity and Ecosystem Services.” In Advances in Ecological Research, edited by J. Dumbrell , R. L. Kordas , and G. Woodward , 43–97. Cambridge, MA: Academic Press Inc. 10.1016/bs.aecr.2016.08.005.

[ece370711-bib-0028] European Environment Agency . 2019. “The European Environment‐State and Outlook 2020.” Knowledge for Transition to a Sustainable Europe. 10.2800/96749.

[ece370711-bib-0029] Finch, T. 2020. “Restoring Farmlands for Food and Nature.” One Earth 3, no. 6: 665–668. 10.1016/j.oneear.2020.11.006.

[ece370711-bib-0030] Gallandt, E. R. , and J. Weiner . 2015. “Crop–Weed Competition.” eLS 2007: 1–9. 10.1002/9780470015902.a0020477.pub2.

[ece370711-bib-0031] Gardein, H. , Y. Fabian , C. Westphal , T. Tscharntke , and A. Hass . 2022. “Ground‐Nesting Bees Prefer Bare Ground Areas on Calcareous Grasslands.” Global Ecology and Conservation 33: e02289. 10.1016/j.gecco.2022.e02289.

[ece370711-bib-0032] Gayer, C. , J. Berger , M. Dieterich , et al. 2021. “Flowering Fields, Organic Farming and Edge Habitats Promote Diversity of Plants and Arthropods on Arable Land.” Journal of Applied Ecology 58, no. 6: 1155–1166. 10.1111/1365-2664.13851.

[ece370711-bib-0033] Goral, F. , and J. Schellenberg . 2024. “Goeveg: Functions for Community Data and Ordinations.” https://cran.r‐project.org/package=goeveg.

[ece370711-bib-0034] Grass, I. , J. Albrecht , F. Jauker , et al. 2020. “Much More Than Bees—Wildflower Plantings Support Highly Diverse Flower‐Visitor Communities From Complex to Structurally Simple Agricultural Landscapes.” Agriculture, Ecosystems & Environment 255: 45–53. 10.1016/j.agee.2016.04.001.

[ece370711-bib-0035] Haaland, C. , R. E. Naisbit , and L. F. Bersier . 2011. “Sown Wildflower Strips for Insect Conservation: A Review.” Insect Conservation and Diversity 4, no. 1: 60–80. 10.1111/j.1752-4598.2010.00098.x.

[ece370711-bib-0036] Habel, J. C. , J. Dengler , M. Janišová , P. Török , C. Wellstein , and M. Wiezik . 2013. “European Grassland Ecosystems: Threatened Hotspots of Biodiversity.” Biodiversity and Conservation 22, no. 10: 2131–2138. 10.1007/s10531-013-0537-x.

[ece370711-bib-0037] Heimer, S. , and W. Nentwig . 1991. Spinnen Mitteleuropas. Ein Bestimmungsbuch. Berlin und Hamburg: Verlag Paul Parey.

[ece370711-bib-0038] Hermoso, V. , S. B. Carvalho , S. Giakoumi , et al. 2022. “The EU Biodiversity Strategy for 2030: Opportunities and Challenges on the Path Towards Biodiversity Recovery.” Environmental Science and Policy 127: 263–271.

[ece370711-bib-0039] Hoffmann, H. , F. Peter , J. D. Herrmann , T. W. Donath , and T. Diekötter . 2021. “Benefits of Wildflower Areas as Overwintering Habitats for Ground‐Dwelling Arthropods Depend on Landscape Structural Complexity.” Agriculture, Ecosystems & Environment 314: 107421. 10.1016/j.agee.2021.107421.

[ece370711-bib-0040] Holl, K. D. , and T. M. Aide . 2010. “When and Where to Actively Restore Ecosystems?” Forest Ecology and Management 261: 1558–1563. 10.1016/j.foreco.2010.07.004.

[ece370711-bib-0042] Horváth, F. , Z. K. Dobolyi , T. Morschhauser , L. Lőkös , L. Karas , and T. Szerdahelyi . 1995. FLÓRA adatbázis 1.2, 267. Vácrátót, Hungary: MTA ÖBKI.

[ece370711-bib-0043] Hothorn, T. , F. Bretz , and P. Westfall . 2008. “Simultaneous Inference in General Parametric Models.” Biometrical Journal 50, no. 3: 346–363. 10.1002/bimj.200810425. 18481363

[ece370711-bib-0044] Hussain, R. I. , M. Brandl , B. Maas , et al. 2021. “Re‐Established Grasslands on Farmland Promote Pollinators More Than predators.” Agriculture, Ecosystems & Environment 319: 107543. 10.1016/j.agee.2021.107543.

[ece370711-bib-0045] Hussain, R. I. , R. Walcher , N. Vogel , B. Krautzer , L. Rasran , and T. Frank . 2022. “Effectiveness of Flowers Strips on insect's Restoration in Intensive Grassland.” Agriculture, Ecosystems & Environment 384: 108436. 10.1016/j.agee.2023.108436.

[ece370711-bib-0046] Hyvönen, T. , E. Huusela , M. Kuussaari , M. Niemi , R. Uusitalo , and V. Nuutinen . 2021. “Aboveground and Belowground Biodiversity Responses to Seed Mixtures and Mowing in a Long‐Term Set‐Aside Experiment.” Agriculture, Ecosystems & Environment 322: 107656. 10.1016/j.agee.2021.107656.

[ece370711-bib-0047] IPBES . 2016. “The Assessment Report of the Intergovernmental Science‐Policy Platform on Biodiversity and Ecosystem Services on Pollinators, Pollination and Food Production.” In Secretariat of the Intergovernmental Science‐Policy Platform on Biodiversity and Ecosystem Services, edited by S. G. Potts , V. L. Imperatriz‐Fonseca , and H. T. Ngo , 552. Bonn, Germany: IPBES. 10.5281/zenodo.3402856.

[ece370711-bib-0048] Kassambara, A. 2023. “Ggpubr: ‘ggplot2’ Based Publication Ready Plots.” https://cran.r‐project.org/package=ggpubr.

[ece370711-bib-0049] Kaur, H. , A. Torma , N. Gallé‐Szpisjak , et al. 2019. “Road Verges Are Important Secondary Habitats for Grassland Arthropods.” Journal of Insect Conservation 23, no. 5–6: 899–907. 10.1007/s10841-019-00171-9.

[ece370711-bib-0050] Kiehl, K. , A. Kirmer , T. W. Donath , L. Rasran , and N. Hölzel . 2010. “Species Introduction in Restoration Projects – Evaluation of Different Techniques for the Establishment of Semi‐Natural Grasslands in Central and Northwestern Europe.” Basic and Applied Ecology 11: 285–299. 10.1016/j.baae.2009.09.002.

[ece370711-bib-0051] Király, G. 2009. “Új magyar füvészkönyv. Magyarország hajtásos növényei. Határozókulcsok. – New Hungarian Herbal.” The Vascular Plants of… Aggteleki Nemzeti Park Igazgatóság.

[ece370711-bib-0052] Kiss, R. , O. Valkó , B. Tóthmérész , and P. Török . 2017. “Seed Bank Research in Central‐European Grasslands – An Overview.” In Seed Banks: Types, Roles and Research, edited by J. Murphy , 1–34. Chapler: Nova Science Publishers Inc.

[ece370711-bib-0053] Klecka, J. , J. Hadrava , P. Biella , and A. Akter . 2018. “Flower Visitation by Hoverflies (Diptera: Syrphidae) in a Temperate Plant‐Pollinator Network.” PeerJ 6: e6025. 10.7717/peerj.6025. 30533311 PMC6282941

[ece370711-bib-0054] Korav, S. , A. Dhaka , R. Singh , and R. Chandramohan . 2018. “A Study on Crop Weed Competition in Field Crops.” Journal of Pharmacognosy and Phytochemistry 7, no. 4: 3235–3240.

[ece370711-bib-0055] Kovács‐Hostyánszki, A. , Z. Elek , K. Balázs , et al. 2013. “Earthworms, Spiders and Bees as Indicators of Habitat Quality and Management in a Low‐Input Farming Region – A Whole Farm Approach.” Ecological Indicators 33: 111–120. 10.1016/j.ecolind.2013.01.033.

[ece370711-bib-0056] Krimmer, E. , E. A. Martin , J. Krauss , A. Holzschuh , and I. Steffan‐Dewenter . 2019. “Size, Age and Surrounding Semi‐Natural Habitats Modulate the Effectiveness of Flower‐Rich Agri‐Environment Schemes to Promote Pollinator Visitation in Crop Fields.” Agriculture, Ecosystems and Environment 284: 106590. 10.1016/j.agee.2019.106590.

[ece370711-bib-0057] Kumar, S. , M. K. Bhowmick , and P. Ray . 2021. “Weeds as Alternate and Alternative Hosts of Crop Pests.” Indian Journal of Weed Science 53, no. 1: 14–29. 10.5958/0974-8164.2021.00002.2.

[ece370711-bib-0058] Li, P. , D. Kleijn , I. Badenhausser , et al. 2020. “The Relative Importance of Green Infrastructure as Refuge Habitat for Pollinators Increases With Local Land‐Use Intensity.” Journal of Applied Ecology 57: 1494–1503. 10.1111/1365-2664.13658.

[ece370711-bib-0059] Maas, B. , M. Brandl , R. I. Hussain , et al. 2021. “Functional Traits Driving Pollinator and Predator Responses to Newly Established Grassland Strips in Agricultural Landscapes.” Journal of Applied Ecology 58, no. 8: 1728–1737. 10.1111/1365-2664.13892.

[ece370711-bib-0060] Malo, J. E. , and F. Suárez . 1995. “Herbivorous Mammals as Seed Dispersers in a Mediterranean Dehesa.” Oecologia 104, no. 2: 246–255. 10.1007/BF00328589. 28307361

[ece370711-bib-0061] Martin, P. A. , N. Ockendon , A. Berthinussen , R. K. Smith , and W. J. Sutherland . 2021. Grassland Conservation: Global Evidence for the Effects of Selected Interventions. Conservation Evidence Series Synopses. Cambridge, UK: University of Cambridge.

[ece370711-bib-0062] McDonald, B. 2007. “Effects of Vegetation Structure on Foliage Dwelling Spider Assemblages in Native and Non‐native Oklahoma Grassland Habitats.” Proceedings of the Oklahoma Academy of Science 87: 85–88.

[ece370711-bib-0063] Mei, Z. , G. A. de Groot , D. Kleijn , et al. 2021. “Flower Availability Drives Effects of Wildflower Strips on Ground‐Dwelling Natural Enemies and Crop Yield.” Agriculture, Ecosystems and Environment 319: 107570. 10.1016/j.agee.2021.107570.

[ece370711-bib-0064] Michalko, R. , S. Pekár , M. Dula , and M. H. Entling . 2019. “Global Patterns in the Biocontrol Efficacy of Spiders: A Meta‐Analysis.” Global Ecology and Biogeography 28, no. 9: 1366–1378. 10.1111/geb.12927.

[ece370711-bib-0065] Miličić, M. , S. Popov , T. Jurca , et al. 2021. “Functional Groups of Hoverflies in Southeast Europe Across Different Vegetation Types.” Entomological Science 24, no. 3: 235–246. 10.1111/ens.12477.

[ece370711-bib-0066] Móczár, M. 1957. “Méhfélék – Apidae. – in: Magyarország Állatvilága (Fauna Hungariae).” XIII/13: 1‐75.

[ece370711-bib-0067] Nentwig, W. , T. Blick , D. Gloor , A. Hänggi , and C. Kropf . 2023. “Araneae: Spiders of Europe.” https://araneae.nmbe.ch.

[ece370711-bib-0068] Nyffeler, M. , and K. D. Sunderland . 2003. “Composition, Abundance and Pest Control Potential of Spider Communities in Agroecosystems: A Comparison of European and US Studies.” Agriculture, Ecosystems & Environment 95, no. 2–3: 579–612. 10.1016/S0167-8809(02)00181-0.

[ece370711-bib-0069] Öckinger, E. , and H. G. Smith . 2007. “Semi‐Natural Grasslands as Population Sources for Pollinating Insects in Agricultural Landscapes.” Journal of Applied Ecology 44: 50–59. 10.1111/j.1365-2664.2006.01250.x.

[ece370711-bib-0070] Oksanen, J. , G. L. Simpson , F. G. Blanchet , et al. 2022. “Vegan: Community Ecology Package.” https://cran.r‐project.org/package=vegan.

[ece370711-bib-0071] Phillips, B. B. , J. M. Bullock , J. L. Osborne , and K. J. Gaston . 2020. “Ecosystem Service Provision by Road Verges.” Journal of Applied Ecology 57, no. 3: 488–501. 10.1111/1365-2664.13556.

[ece370711-bib-0072] Plath, E. , T. Rischen , T. Mohr , and K. Fischer . 2021. “Biodiversity in Agricultural Landscapes: Grassy Field Margins and Semi‐Natural Fragments Both Foster Spider Diversity and Body Size.” Agriculture, Ecosystems & Environment 316: 107457. 10.1016/j.agee.2021.107457.

[ece370711-bib-0073] Roberts, D. W. 2023. “Labdsv: Ordination and Multivariate Analysis for Ecology.” https://cran.r‐project.org/package=labdsv.

[ece370711-bib-0074] Rodríguez‐Gasol, N. , G. Alins , E. R. Veronesi , and S. Wratten . 2020. “The Ecology of Predatory Hoverflies as Ecosystem‐Service Providers in Agricultural Systems.” Biological Control 151: 104405. 10.1016/j.biocontrol.2020.104405.

[ece370711-bib-0075] Samu, F. , and C. Szinetár . 2002. “On the Nature of Agrobiont Spiders.” Journal of Arachnology 30, no. 2: 389–402.

[ece370711-bib-0076] Savage, J. , B. A. Woodcock , J. M. Bullock , M. Nowakowski , J. R. B. Tallowin , and R. F. Pywell . 2021. “Management to Support Multiple Ecosystem Services From Productive Grasslands.” Sustainability (Switzerland) 13, no. 11: 6263. 10.3390/su13116263.

[ece370711-bib-0077] Schaffers, A. P. , I. P. Raemakers , K. V. Sýkora , and C. J. F. Ter Braak . 2008. “Arthropod Assemblages Are Best Predicted by Plant Species Composition.” Ecology 89, no. 3: 782–794. 10.1890/07-0361.1. 18459341

[ece370711-bib-0078] Schneider, G. , J. Krauss , and I. Steffan‐Dewenter . 2013. “Predation Rates on Semi‐Natural Grasslands Depend on Adjacent Habitat Type.” Basic and Applied Ecology 14, no. 7: 614–621. 10.1016/j.baae.2013.08.008.

[ece370711-bib-0079] Schroeder, P. J. , and D. G. Jenkins . 2018. “How Robust Are Popular Beta Diversity Indices to Sampling Error?” Ecosphere 9, no. 2: e02100. 10.1002/ecs2.2100.

[ece370711-bib-0080] Snow, G. 2024. “TeachingDemos: Demonstrations for Teaching and Learning.” https://cran.r‐project.org/package=TeachingDemos.

[ece370711-bib-0081] Speight MCD . 2020. StN key for the Identification of the Genera of European Syrphidae (Diptera) 2020. Syrph the Net, the Database of European Syrphidae, Vol 105, 46. Dublin: Syrph the Net Publications.

[ece370711-bib-0082] Sutherland, J. P. , M. S. Sullivan , and G. M. Poppy . 2001. “Distribution and Abundance of Aphidophagous Hoverflies (Diptera: Syrphidae) in Wildflower Patches and Field Margin Habitats.” Agricultural and Forest Entomology 3, no. 1: 57–64. 10.1046/j.1461-9563.2001.00090.x.

[ece370711-bib-0083] Threadgill, K. R. D. , C. J. McClean , J. A. Hodgson , N. Jones , and J. K. Hill . 2020. “Agri‐Environment Conservation Set‐Asides Have Co‐Benefits for Connectivity.” Ecography 43, no. 10: 1435–1447. 10.1111/ecog.05127.

[ece370711-bib-0084] Török, E. , S. Zieger , J. Rosenthal , et al. 2021. “Organic Farming Supports Lower Pest Infestation, but Less Natural Enemies Than Flower Strips.” Journal of Applied Ecology 58, no. 10: 2277–2286. 10.1111/1365-2664.13946.

[ece370711-bib-0085] Török, P. , E. Vida , B. Deák , S. Lengyel , and B. Tóthmérész . 2011. “Grassland Restoration on Former Croplands in Europe: An Assessment of Applicability of Techniques and Costs.” Biodiversity and Conservation 20, no. 11: 2311–2332. 10.1007/s10531-011-9992-4.

[ece370711-bib-0086] Tóth, S. 2011. “Magyarország zengőlégy faunája (Diptera: Syrphidae) Hoverfly Fauna of Hungary. – E‐Acta Naturalia Pannonica.” Supplementum 1: 5–408.

[ece370711-bib-0087] United Nations General Assembly . 2019. “73/284. United Nations Decade on Ecosystem Restoration (2021‐2030), The Dingo Barrier Fence: Presenting the Case to Decommission the world's Longest Environmental Barrier in the United Nations Decade on Ecosystem Restoration 2021–2030.” 10.1007/s42977-021-00106-z 34807433

[ece370711-bib-0088] Vujanović, D. , G. Losapio , M. Mészáros , et al. 2023. “Forest and Grassland Habitats Support Pollinator Diversity More Than Wildflowers and Sunflower Monoculture.” Ecological Entomology 48, no. 4: 421–432. 10.1111/een.13234.

[ece370711-bib-0089] Wen, A. , K. J. Elgersma , M. E. Sherrard , L. L. Jackson , J. Meissen , and M. C. Myers . 2022. “Wild Bee Visitors and Their Association With Sown and Unsown Floral Resources in Reconstructed Pollinator Habitats Within an Agriculture Landscape.” Insect Conservation and Diversity 15, no. 1: 102–113. 10.1111/icad.12539.

[ece370711-bib-0090] Wickham, H. 2016. ggplot2: Elegant Graphics for Data Analysis. New York: Springer‐Verlag. https://ggplot2.tidyverse.org.

[ece370711-bib-0091] Wickham, H. , M. Averick , J. Bryan , et al. 2019. “Welcome to the {Tidyverse}.” Journal of Open Source Software 4, no. 43: 1686. 10.21105/joss.01686.

[ece370711-bib-0092] Wickham, H. , R. François , L. Henry , and K. Müller . 2022. “Dplyr: A Grammar of Data Manipulation.” https://cran.r‐project.org/package=dplyr.

